# Crossing scales, crossing disciplines: collective motion and collective action in the Global Commons^†^

**DOI:** 10.1098/rstb.2009.0197

**Published:** 2010-01-12

**Authors:** Simon Levin

**Affiliations:** 1 Department of Ecology and Evolutionary Biology, Princeton University, Princeton, NJ, USA; 2 Beijer Institute for Ecological Economics, Stockholm, Sweden; 3 Resources for the Future, Washington DC, USA

**Keywords:** collective motion, cooperation, social norms, Global Commons

## Abstract

Two conflicting tendencies can be seen throughout the biological world: individuality and collective behaviour. Natural selection operates on differences among individuals, rewarding those who perform better. Nonetheless, even within this milieu, cooperation arises, and the repeated emergence of multicellularity is the most striking example. The same tendencies are played out at higher levels, as individuals cooperate in groups, which compete with other such groups. Many of our environmental and other global problems can be traced to such conflicts, and to the unwillingness of individual agents to take account of the greater good. One of the great challenges in achieving sustainability will be in understanding the basis of cooperation, and in taking multicellularity to yet a higher level, finding the pathways to the level of cooperation that is the only hope for the preservation of the planet.

## 1. Natural selection and evolution: bringing order to the biological world

The great biologist, Theodosius Dobzhansky, wrote that ‘Nothing in biology makes sense except in the light of evolution’ (Dobzhansky 1964, 1973). Without question, the theory of evolution through natural selection is the fundamental organizing theme in biology, helping to explain the emergence of phenomenal complexity, the diversity of organisms and how those organisms become arranged and inter-related in biological communities and ecosystems.

Natural selection is a force, much like gravity (or, in Wallace's (1858) terminology, ‘a centrifugal governor’). That we still do not understand all the implications of natural selection, and its interactions with other influences, should be no more surprising than that we do not fully understand all of the implications of how gravity works in situations where diverse bodies exert interacting gravitational forces. In complex ecological communities, natural selection is operating at many levels simultaneously, and the consequences may be impossible to predict, and very sensitive to ‘frozen accidents’, random events that become fixed.

As an explanatory tool, however, natural selection helps peel away the mysteries of the biological world. The explanatory power of natural selection is in its simplicity: it is little more than a filter, acting on random variation generated by chance events and itself varying with secular changes in the physical, chemical and biotic environments. Indeed, the basic principles have proved useful in mathematical optimization problems that are too difficult for explicit solution, and in the practical application to design problems as diverse as how to synthesize effective drugs and efficient jet engines.

Charles Darwin (1809–1882) is, properly, recognized as the father of the theory of natural selection, although credit must be given to the influence of Thomas Malthus, as well to Darwin's friends, the geologist Charles Lyell (1797–1875), who influenced Darwin greatly, and Alfred Russel Wallace (1823–1913), who independently proposed essentially the same theory of natural selection in 1858 and thereby induced Darwin to publish his works earlier than he would have otherwise (Wallace 1858).

Darwin, the naturalist on the five-year voyage of HMS *Beagle*, captained by Robert FitzRoy, was not only able to draw pattern from his observations in diverse climes, but to infer process as well. The idea is a simple one: chance events continually produce new variants, some of which are better suited in a changing environment than the current common type, and they leave more offspring, which themselves reproduce. Eventually, such a new type will replace the current type, only to be replaced itself in the future as the environment changes further, and yet other types arise. This is a never-ending tale, the inescapable consequence of the simple rule. Different conditions as well as initial evolutionary accidents produce different patterns, allowing the rich diversity of organisms we see today. But change is inevitable, and even the human species evolves, and will continue to evolve. Some changes in all species are minor; others have major fitness consequences, for example affecting susceptibility to disease and suitability to changing climatic conditions. At the broader level of multiple populations, selection pressures can be sufficiently different leading to speciation events.

Natural selection can be strong and lead to rapid evolutionary change, as in the evolution of bacteria, or it can be weak. But, even at its weakest, it is a patient mechanism; and, given enough time, even weak selection can give rise to fantastic diversity, and mystifying complexity. One of the keys to the evolution of that complexity is cooperation among diverse types of organisms, which makes essential an understanding of how cooperation arises. As we will see in later sections, cooperation was a challenge for the classical theory, but recent and current research is opening the curtains that have obscured clarity.

## 2. Challenges for the classical theory

The theory of evolution through natural selection is, as already discussed, a powerful tool for explaining how change takes place, and how organisms are continually reshaped in changing environments. It is sometimes misconstrued, however, as leading to optimization or perfection, and in its most naive form might be thought to lead to a single best type. Natural selection, in its simplest clothes, is, after all, a homogenizing process; so how does diversity arise in the first place, and how is it maintained?

Climatic and other conditions across the globe are highly variable, on many different scales, creating uncountable opportunities for specialization. The existence of such variation can select for mechanisms, like mutation and recombination, that facilitate the ability of genomes to exploit it. The homogenizing effects of stabilizing selection are thereby balanced by processes that restore variation, thereby maintaining the adaptive capacity of populations.

Micro-organisms are nimble and short-lived, and can take advantage of opportunities that we cannot even measure. The first organisms to evolve when the Earth began were simple micro-organisms, probably under the Earth's surface, or in its oceans. Over billions of years, their cumulative effects produced increasing levels of oxygen in our atmosphere, and gradually led to the evolution of a greater diversity of organisms, with each layer of complexity building on what had already evolved.

Some organisms became more and more complex, involving many kinds of cells, with different basic functions. Humans, of course, are highly differentiated, with specific tissues and organs for performing diverse tasks; but even bacteria have specialized parts, such as the organelles that serve as propellers to drive them through their fluid environments. Development of organs from groups of tissues is universal in plants and animals. They are all *multicellular*, meaning they have more than one cell, including ones differentiated for specific tasks.

### (a) Cooperation, complexity and multicellularity

Complexity can arise by the familiar processes of mutation and recombination; however, this process is inadequate to explain the degree of complexity we see today. Other increases in complexity have occurred when organisms have simply engulfed other organisms that could perform particular functions, and then made them part of a new whole. Lynn Margulis (1981), for example, proposed that mitochondria, which are crucial parts of eukaryotic cells, arose by a process of endocytosis (ingestion) of aerobic bacteria (which require oxygen) by anaerobic bacteria (normally unable to live in oxygen-rich environments), creating a permanent partnership. Similarly, she developed the notion that chloroplasts, which allow plants and eukaryotic algae to photosynthesize, emerged when photosynthetic bacteria were ingested (see also Raven 1970).

These examples of complexification arose from an initial mutualism between different species. More generally, there are numerous examples of mutualism between species in which the individual reproductive identities have been maintained. Many sea anemones, for example, incorporate single-celled algae, which can photosynthesize and provide oxygen, glucose and other foods to the anemones. Fungi and algae also form symbiotic associations, *lichens*, deriving the nutritional benefits of algal photosynthesis, and the algae, among other things, benefit from water retention and absorption of nutrients by the fungi.

We are multicellular; and, of course, so are most organisms that we can see without a microscope. Multicellular organisms are the most complex in nature, and the multiple origins of this complexity have provided another challenge for evolutionary theory (Bonner 1993, 2000). Many, if not most, examples of multicellularity probably arose simply from cooperation among members of the same species. This is, for good reasons, termed the *colonial* theory, since it involves individuals ‘learning’ first to live in colonies, before those colonies became integrated wholes over evolutionary time. Biologists have studied colony formation in systems ranging from bacterial mats and slime molds, to coral reefs and beehives, from swarms of insects to schools of fish, from herds of bison and wildebeest to primate troops. In human societies, mechanisms of cooperation are well established, but unfortunately limited in scope. We cooperate in small groups, but often largely for the purpose of competition and combat with other groups. Unless and until we can learn to get beyond that, and to achieve a multicellular state of mind that encompasses all of humanity, our chances for global survival are diminished.

## 3. The emergence of cooperation and competition at higher levels of organization

There is much to be learned about cooperation among humans by studying cooperation among other organisms; but we must recognize that with each new case, new mechanisms become important. Humans have highly evolved capabilities of calculation and prediction, and high levels of communication using language with syntax. Imitation and evolutionarily refined responses to simple cues remain important, but the human social context introduces complexity that can foster cooperation at certain levels of organization, and impede it at others.

Cooperation is at the root of multicellularity, and cooperation is central to addressing our common problems. At first blush, cooperation seems exactly the opposite of what natural selection—the survival of the fittest—is about. Yet, every living multicellular organism represents a group of highly altruistic components, and we see cooperation, indeed even altruism, among members of many other species. How does this apparent anomaly occur? How does cooperation arise in communities of diverse organisms; and why and when does natural selection not only allow cooperation, but even reinforce it?

In the haplodiploid insects—the bees, the ants and the wasps—most females are sterile workers, giving up their own fitness for the good of the colony. Darwin (1859) was puzzled by these situations; in Chapter VIII of *On the origin of species*, he wrote that this paradox ‘at first appeared to me insuperable, and actually fatal to the whole theory.’ But Darwin concluded that, although this was ‘by far the most serious special difficulty that my theory has encountered’, nevertheless, natural selection could fully account for such extreme altruism, provided selection was operating at a higher level of organization—‘the family’. In this, he anticipated later solutions to the problem, but without knowing of the more specific genetic explanations that would come nearly a century later, or the inevitable debates about group selection and when it could operate (Lehmann *et al*. 2007). Darwin, not surprisingly, also recognized the relevance to the central theme of this paper, cooperation among humans: ‘We can see how useful their production may have been to a social community of ants, on the same principle that the division of labour is useful to civilized man’. I will return to this theme shortly.

The influential British biologist, J.B.S. Haldane, reportedly explained apparent altruism most succinctly, when he said ‘I'd lay down my life for two brothers or eight cousins’. What Haldane meant simply was that, since he shared half his genes with each brother, two brothers together would carry as many of his genes as did he himself. Similarly, since children share half their parents' genes, two first cousins share one-eighth of their genes with one another, so eight cousins are equivalent to two brothers, or equivalent to Haldane himself.

Nowhere is such altruistic behaviour between multicellular individuals more widespread than in the haplodiploid insects. Why should that be? Haplodiploid means that males are haploid—they arise from unfertilized eggs, so only get the genes that their mothers contribute. Females are diploid—they get half their genes from their fathers, and half from their mothers. Because all the sperm produced by a male are genetically identical, full sisters share all the genes that they received from their fathers, and half the genes that they received from their mothers. Therefore, on average, sisters born of the same father are identical in three-fourths of their genes. Compare that with the fact that Haldane's sister, Naomi Mitchison, would have shared only one-half of her genes with any sisters, or with J.B.S. himself. For a haplodiploid insect, then, there is even greater incentive to be altruistic. The late evolutionary biologist William D. Hamilton worked out the esoteric mathematical details of this idea, later called ‘kin selection’, in a pair of fundamentally important papers. (Hamilton 1964*a*,*b*).

Kin selection certainly provides insight into why individuals help their kin, but cooperative behaviour among non-kin is widespread in animal societies. The simplest explanation for such behaviour is called ‘reciprocal altruism’, in which individuals engage in altruistic acts in the expectation that the favour will be returned at some time in the future. We are, of course, very familiar with such behaviour in human societies, and reinforce it by forming friendship bonds, or other such groupings. However, similar reciprocal cooperation can occur when individuals interact more diffusively within large groups, as long as there is a reasonable expectation that the beneficiaries will exhibit similar altruistic tendencies. The difficulty is that, as groups get large, that expectation goes down. Groups split into smaller groups in which reciprocal altruism can be maintained, and these groups may then interact competitively and aggressively with other groups.

As our societies have grown larger, the simplest forms of reciprocal altruism have broken down. Over our history, in culture after culture, small tribal groups, held together by reciprocal altruism, have realized that there are benefits to be gained by banding together with other such tribal groups, often in order to compete with yet other such larger groups. At this level, though, reciprocal altruism is not enough, and common rules must be agreed upon, together with punishments for violating those rules, often called ‘social norms’. Those rule systems have become formalized as laws and customs, holding together societies and religions. Recent experiments (see for example Fehr & Schmidt 1999; Fehr & Gaechter 2000) have shown that, over millennia of cultural evolution, individuals have internalized such rules, as well as the willingness to punish defectors, even at cost to the punisher. In the case of religions, the threat of punishment can be especially effective, because it is reserved for a future time, after death, making it impossible to judge whether it really will be imposed.

Understanding social norms, and cultural behaviour more generally, is a research undertaking of deep theoretical and empirical importance. This is not a new topic, and a theoretical foundation has been built over the past quarter century (see for example Wilson 1975; Cavalli-Sforza & Feldman 1981; Axelrod 1984; Boyd & Richerson 1985; Ehrlich 2000; Wilson 2002; Bowles & Gintis 2004; Durrett & Levin 2005; Nowak & Sigmund 2007). Increasingly, however, it has become clear that if we are to find solutions to global environmental and economic problems, we will need increased emphasis on such efforts to understand how to achieve cooperation among nations (Levin 1999; Ehrlich & Kennedy 2005; Ehrlich & Levin 2005). In the areas of environment and the economy, existing institutions are insufficient to prevent tragedies of the commons, and the time is short to learn how to establish them (Walker *et al*. 2009).

Social norms can play highly beneficial roles within our societies, prohibiting socially damaging behaviours like theft, adultery and murder, and encouraging respect for our environment. But social norms equally can be damaging, leading to the maintenance of discriminatory caste systems, or leading to overconsumption and the exploitation of environmental resources. My Princeton colleague, Kwame Anthony Appiah, in his book *The Ethics of Identity*, confronts this dilemma by asking, ‘Is culture a good?’ (Appiah 2007). This mandates a search for values and social norms that involve mutual respect, as well as respect for our diverse pasts and common future.

A second challenge, to which I return in the last section, is that groups with common interests and common norms often owe their existence to the fact that these coalitions provide advantages in competition with other groups. Once those external threats disappear, the groups have less reason to exist, and are likely to fragment and turn to internal conflict. Our challenge is to find ways to maintain cooperation in the common good even without the mechanism of competition and conflict with other groups.

## 4. The global commons

Recent years have seen a growing awareness of the deteriorating state of our environment. Our consumptive patterns have led to a toxification of our environment, accelerated climate change, and depleted biodiversity and other natural resources; growing conflicts among peoples are in no small way linked to these changes. Though we are the cause, we stand as observers, seemingly powerless to reverse the effects of our own actions. Why?

The problem is that we live in a Global Commons, in which we bear neither the full costs, nor the full benefits, of our actions. We feel individually only marginally responsible for the world's problems, and that our own sacrifices will do little to improve the situation. Economic markets, counted on by many to resolve problems of shortages, do not work effectively because they ignore the social costs, the externalities, and shortchange future generations by imposing high discount rates.

When we lived in small groups, and when our effects were regionally limited, this was less of a problem. Reciprocal altruism had a chance to work, even without the stimulus of intertribal conflict. As groups became larger, however, globalization increased, and our problems assumed larger and even global scales, while their solutions faded further into the distance. Intra-group cooperation remained strong, and nationalism increased; but conflicts among nations made matters worse.

Even in small societies, problems of the commons require work to resolve. Lloyd (1833), an Oxford political economist, puzzled about the stunted status of cattle on common pasture areas in England: ‘Why are the cattle on a common so puny and stunted? Why is the common itself so bare-worn, and cropped so differently from the adjoining inclosures?’ The answer, he concluded, was in a microcosm of the problems we face today. As Hardin (1968) explained Lloyd's arguments more than a century later, the commons environment creates a conflict in which there is benefit to the individual to overexploit, but cost to society. ‘Therein is the tragedy. Each man is locked into a system that compels him to increase his herd without limit—in a world that is limited. Ruin is the destination towards which all men rush, each pursuing his own best interest in a society that believes in the freedom of the commons. Freedom in a commons brings ruin to all’. Hardin called this the ‘Tragedy of the Commons’.

Lloyd's insights caused a sea change in Hardin's thinking, in which he realized that the conventional economic thinking fell short of providing solutions. ‘With Adam Smith's work as a model, I had assumed that the sum of separate ego-serving decisions would be the best possible one for the population as a whole. But presently I discovered that I agreed much more with William Forster Lloyd's conclusions… Citing what happened to pasturelands left open to many herds of cattle, Lloyd pointed out that, with a resource available to all, the greediest herdsmen would gain—for a while. But mutual ruin was just around the corner. As demand grew in step with population (while supply remained fixed), a time would come when the herdsmen…would be trapped by their own competitive impulses. The unmanaged commons would be ruined by overgrazing; competitive individualism would be helpless to prevent the social disaster.

So must it also be, I realized, with growing human populations when there is a limit to available resources. … It was so in Lloyd's day; it is even more so today.’

These are the challenges we still face today, but they are greater and more compelling than ever before. These are the challenges that require we find a collective mission, built on cooperation and common purpose. The scientific study of cooperation has a central role to play in achieving that cooperation, and must involve multidisciplinary approaches, from evolutionary theory to economics. This has, for example, led the European Science Foundation to launch a recent research programme on ‘Evolutionary Perspectives on Cooperation and Trading’. It has never been more evident than in the desperate international efforts to find coordinated solutions to the current global economic meltdown.

## 5. Achieving cooperation at the highest levels

Cooperation is widespread in the biological world, especially in human societies. Bacteria signal one another by exuding chemicals, and exchange mutual favours (Wingreen & Levin 2006; Nadell *et al*. 2008*a*,*b*). Amoebae organize themselves into slime molds, insects into swarms, birds into flocks, fish into schools, ungulates into herds. Primates have the most highly developed social organizations of unrelated individuals, relying on highly developed cultural practices to maintain the integrity of their societies.

But the tribes and societies and cultures we build become devices for conflict among groups, and too often it is that conflict and competition that strengthens the membership bonds. When groups come together, it is often because there is a common enemy. How can we get beyond this in achieving the survival of our species, and of our planet?

We must recognize that we have a common enemy, and that enemy is the extinction that awaits us if we do not change our ways. It is war and pollution, it is biodiversity loss and climate change, it is all the things that threaten the quality of our life, as well as our survival. The sooner we acknowledge this common threat, the sooner we can achieve the cooperation that will bond us all together.

However, as discussed earlier, cooperation becomes more difficult to sustain as group size increases. Among humans, this constraint has been met through informal agreements, what Hardin (1968) called ‘mutual coercion, mutually agreed upon’, which ultimately may become formalized into traditions, customs, rules and laws. These agreements may provide individuals within the groups benefits they would not have if solitary, or in smaller groups; but they also can become sustained even if they do not confer benefits, simply because defectors are punished. Societies thereby become locked into patterns of behaviour—social norms—that are curiously resistant to change over long periods of time, sometimes centuries; but, just as mysteriously, those patterns of behaviour can collapse suddenly, giving rise to rapid change in collective behaviour. A famous example involves the practice of foot-binding in China, but changes also occur rapidly in patterns of dress, in attitudes towards smoking and with regard to the role of women in many societies. These transitions are driven by contagious behaviours, as individuals imitate other individuals; but certain individuals occupy positions of key influence, and can trigger such societal shifts in behaviour. This is true not only in human societies, but in almost all animal groupings. The phenomenon has been observed in experimental situations, as well as in theoretical models that explore the relationship between leaders and followers (Couzin *et al*. 2005). Couzin and co-workers are interested in the mechanisms that result in the coordination of schools and swarms in fish, birds and other species; but lessons are apparent for understanding collective decision-making in humans. The remarkable insight is that a very small number of leaders can cause shifts in behaviours of large groups of individuals, and lead them to coordinate their behaviours. Like a collection of self-stimulating pendulums, they easily become synchronized in their actions, a powerful collective force rather than a random collection without communality. This perhaps should not surprise us based on our understanding of human groups. The problem is that those few influential individuals can as easily lead in bad directions as in good; the models do not discriminate. Our challenge thus is to find the leaders who can take us down good paths. There are those that argue that we cannot hope to achieve the necessary changes in our societies without laws and punishments imposed from the top down. That may or may not be true, but those top-down constraints will be difficult or impossible to impose if they do not reflect popular movements that lay the groundwork for them. Changing social norms creates the milieu in which bold leadership is possible; it is where we must begin. This imperative makes the strong case for the multidisciplinary research efforts mentioned earlier, or for collaboration among natural scientists and social scientists, theoreticians and experimentalists. Cooperation among such diverse researchers will be the key experimental test of whether we can achieve the cooperation necessary to achieve a sustainable future.
